# FCMDAP: using miRNA family and cluster information to improve the prediction accuracy of disease related miRNAs

**DOI:** 10.1186/s12918-019-0696-9

**Published:** 2019-04-05

**Authors:** Xiaoying Li, Yaping Lin, Changlong Gu, Jialiang Yang

**Affiliations:** 1 College of Computer Science and Electronic Engineering, Hunan University, Changsha, 410082 Hunan China; 2 Department of Genetics and Genomic Sciences, Icahn School of Medicine at Mount Sinai, New York, NY 10029 USA

**Keywords:** Disease-related miRNA, Leave-one-out cross validation, miRNA family information, miRNA cluster information, Nearest neighbor recommendation algorithm

## Abstract

**Background:**

Biological experiments have confirmed the association between miRNAs and various diseases. However, such experiments are costly and time consuming. Computational methods help select potential disease-related miRNAs to improve the efficiency of biological experiments.

**Methods:**

In this work, we develop a novel method using multiple types of data to calculate miRNA and disease similarity based on mutual information, and add miRNA family and cluster information to predict human disease-related miRNAs (FCMDAP). This method not only depends on known miRNA-diseases associations but also accurately measures miRNA and disease similarity and resolves the problem of overestimation. FCMDAP uses the k most similar neighbor recommendation algorithm to predict the association score between miRNA and disease. Information about miRNA cluster is also used to improve prediction accuracy.

**Result:**

FCMDAP achieves an average AUC of 0.9165 based on leave-one-out cross validation. Results confirm the 100, 98 and 96% of the top 50 predicted miRNAs reported in case studies on colorectal, lung, and pancreatic neoplasms. FCMDAP also exhibits satisfactory performance in predicting diseases without any related miRNAs and miRNAs without any related diseases.

**Conclusions:**

In this study, we present a computational method FCMDAP to improve the prediction accuracy of disease related miRNAs. FCMDAP could be an effective tool for further biological experiments.

**Electronic supplementary material:**

The online version of this article (10.1186/s12918-019-0696-9) contains supplementary material, which is available to authorized users.

## Background

MicroRNAs (miRNAs) are small endogenous non-coding RNAs with length of about 22 nt and can regulate gene expression mainly through post-transcription [1]. The latest version of miRBase consists of 1881 human miRNAs, and most of them regulate more than 60% of human protein-coding genes. miRNAs regulate target genes through biological processes, such as cell growth, proliferation, differentiation and apoptosis. miRNAs play a critical role in the development of various diseases including cancers [2]. Takamizawa et al. [3] found that the expression level of let-7 decreases in lung neoplasms in vivo and in vitro, resulting in shortened post-operative survival of the patients. Moreover, let-7 is a potential therapeutic miRNA for prevention of tumorigenesis. Lung neoplasms are characterized by several key oncogene mutations, including p53, RAS, and MYC; some of which may be directly related to the decreased expression of let-7 and may be inhibited by introducing this miRNA [3]. miRNAs can be used as biomarkers to identify cancer tissure origin of unknown primary origin [4, 5]. Therefore, identification of disease-related miRNAs would benefit research on pathogenesis and diagnosis.

Many disease-related miRNAs have been identified through biological experiments. Researchers have collected data from existing literature to build miRNA-related databases, such as miRBase [6], miRGen [7], miRTarBase [8], miRWalk [9], microRNA.org [10], miRCancer [11], HMDD [12], miR2Disease [13], dbDEMC [14], and PhenomiR [15]. These databases provide solid data foundation for study of miRNAs. However, methodologies for screening of miRNA-disease associations are costly and time consuming. In this regard, computational methods are used to predict miRNAs that are most likely associated with a disease and provide experimental targets for biological experiments to save cost and time.

Computational methods are classified into two main categories, namely, network-based methods and machine-learning-based methods [16]. Network-based methods predict unknown miRNA-disease associations by constructing different computational models using miRNAs and disease-related data resources to construct miRNA and disease similarity networks [17]; the obtained data are then combined with experimentally validated (or known) miRNA-disease networks. Jiang et al. [18] proposed a miRNA-prediction algorithm for the hypergeometric distribution scoring system, and the scores are ranked to select candidate disease- related miRNAs. Chen et al. [19] proposed WBSMDA method, which integrates the With-Score of miRNA and diseases similarity and the Between-Score of unknown miRNA-disease associations to predict potential miRNA-disease associations. However, the two methods make assumptions about probability distribution, and their prediction performances will be affected when the data resources are inconsistent with the assumptions. Xuan et al. [20] proposed HDMP method by considering weighted *k* most similar neighboring miRNAs and combining miRNA functional similarity to predict miRNAs associated with human diseases. RWRMDA [21] and MIDP [22] methods use random walk to calculate similarity of miRNAs and diseases. However, these methods cannot predict related miRNAs for diseases without any related miRNAs or new diseases (isolated diseases). Zou et al. [23] proposed KATZ to calculate the prediction score of different walking lengths between miRNAs and diseases through social network analysis. However, the performance of KATZ is poor because the known associations are sparse. KATZ also cannot predict related diseases for miRNAs without known related diseases or new miRNAs (isolated miRNAs). However, KATZ cannot be used to predict related miRNAs for isolated diseases.NCPMDA [24] develops network consistency projection to calculate potential miRNA–disease association score from miRNA and disease vector space projection scores. Li et al. [25] proposed a network similarity integration method (NSIM) for predicting potential miRNA-disease associations. However, NSIM are overly dependent on known miRNA-disease associations. HGIMDA [26] utilizes a heterogeneous graph iterative algorithm based on known miRNA–disease associations to predict miRNA–disease associations. However, HGIMDA is difficult to use in selecting parameters.

Machine learning-based methods aim to predict reliable miRNA-disease association by extracting effective features or solving specific optimization problems by using powerful machine-learning algorithms. Xu et al. [27] built a support vector machine (SVM) classifier by using four topological features based on the miRNA target-dysregulated network to predict potential miRNAs related to prostate cancer. The main disadvantage of Xu’s method is the impossibility to obtain negative samples, thereby decreasing the prediction performance. Chen and Yan [28] proposed RLSMDA method that uses regularized least squares to predict miRNA-disease associations. This method is based on semi-supervised learning and avoid using negative samples but adjust parameters intricately. Li et al. [29] proposed MCMDA method using the matrix completion algorithm. Luo et al. [30] proposed CPTL method using the transduction learning collective prediction model to predict miRNA-disease associations. However, these methods cannot be applied to predict potential miRNAs for isolated diseases.

These above methods use only a single piece of information related to miRNAs or diseases, such as association of miRNAs and diseases verified by biological experiments, resulting in overestimation [31]. Therefore, researchers have investigated different types of miRNA- and disease-related a priori biological information to construct miRNA–disease associations through intermediaries. For example, Mørk et al. [32] developed a miRNA–protein–disease heterogeneity-related network, namely, miRPD, which uses protein-related associations as a bridge to link miRNAs and diseases. However, the prediction accuracy of miRPD is unsatisfactory because of its high false positive/negative rates. Xu et al. [33] used the network of interactions between miRNAs and target genes derived from matched miRNA and mRNA expression data and the network of interactions between specific miRNAs and diseases to sequence and identify miRNAs most likely associated with multiple diseases. Liu et al. [31] integrated miRNA-target gene and miRNA-lncRNA multiple data sources, established disease and miRNA similarity subnets, and predicted miRNA-disease associations in heterogeneous networks by using random walk with restart. Zeng et al. [34] used gene functional information, four main parameters of miRNAs and miRNA-disease associations to construct a bilayer networks. Then they used structural consistency as an indicator to estimate the link predictability of the bilayer networks, and used structural perturbation method (SPM) to predict potential miRNA-disease associations. SRMDAP [35] builds miRNA and disease similarity subnetworks by using the SimRank algorithm and density-based clustering recommender model based on known miRNA-mRNA interaction data, disease-gene data, and miRNA-disease association data. However, these methods lead to incomplete calculation of similarity and low prediction accuracy.

In our work, we propose a novel computational method, namely, FCMDAP, by using miRNA family and cluster information to improve the prediction accuracy of disease-related miRNAs. FCMDAP uses information entropy and mutual information (MI) to measure similarity between miRNAs based on miRNA–mRNA interaction and adds miRNA family information to reconstruct a miRNA similarity network. FCMDAP obtains functional similarity between diseases based on disease–gene interaction and semantic similarity between diseases based on disease directed acyclic graph (DAG). FCMDAP then integrates functional and semantic similarity to disease similarity. Based on the *k*-most similar neighboring recommendation algorithm, FCMDAP uses experimentally verified miRNA–disease association, miRNA similarity, and cluster information to predict potential miRNA–disease associations in miRNA space. FCMDAP also uses experimentally verified miRNA–disease association and disease similarity to predict potential miRNA–disease associations in disease space. The two predicted association scores are linearly integrated together. We implemented leave-one-out cross validation (LOOCV) and achieved AUC of 0.9165. Analysis of miRCancer, dbDEMC, or PhenomiR databases, confirmed the 50, 49, and 48 of top 50 predicted miRNAs in case studies of colorectal, lung, and pancreatic neoplasms, respectively. The average AUC values of FCMDAP to predict isolated diseases and miRNAs were 0.8417 and 0.8944, respectively. For isolated lung neoplasms, all of the top 50 predicted miRNAs were confirmed. For isolated hsa-mir-93, 9 of the top 10 diseases were confirmed. In conclusion, FCMDAP outperforms other methods.

## Materials

### Data

Data used in FCMDAP are obtained from five data sets:

1. experimentally verified miRNA-disease related data from HMDD v2.0 database (http://www.cuilab.cn/hmdd, Jun-14-2014 Version) [12]. After filtering invalid data with disease name error or wrong miRNA name and removing redundant miRNA-disease associations, we obtained 5048 experimentally verified miRNA-disease associations including 475 miRNAs and 334 diseases as the benchmark dataset [see Additional file 1]. We use *M* = {*m*_1_, *m*_2_, ⋯, *m*_*nm*_} to represent the miRNA set and *D* = {*d*_1_, *d*_2_, ⋯, *d*_*nd*_} to represent the disease set, where *nm* is the number of miRNAs, and *nd* is the number of diseases. We also use the matrix *AS* to represent the known association of miRNAs and diseases. When miRNA *i* associates with disease *j*, *AS*(*i*, *j*) is 1. Otherwise, *AS*(*i*, *j*) is 0.
2. experimentally verified miRNA-mRNA interactions from miRTarBase database (http://mirtarbase.mbc.nctu.edu.tw/, Release 6.0: Sept-15-2015) [36]. We use these data to measure functional similarity of miRNAs.
3. experimentally verified disease-gene interaction from DisGeNET database (http://www.disgenet.org, Release 4.0: Oct-2016) [37]. We use these data to measure functional similarity of diseases.
4. data on the relationship of various disease from the MeSH (http://www.nlm.nih.gov/, 2017 Version) descriptor of Category C, which are descripted as DAG. We use these data to measure semantic similarity of diseases.
5. information of the family and cluster of human miRNAs from miRBase (http://www.mirbase.org, Release 21) [6]. We established the miRNA family information matrix *FAM* for the 475 miRNAs in the benchmark. *FAM*(*i*, *j*) = 1 if miRNA *i* and *j* are in the same family; otherwise, *FAM*(*i*, *j*) = 0. We also established the miRNA cluster information matrix *CLU* for 475 miRNAs. *CLU*(*i*, *j*) = 1 if the distance between miRNA *i* and *j* is less than 20 kb and we consider the two miRNAs in the same cluster; otherwise, *CLU*(*i*, *j*) = 0.

### miRNA similarity network

Information entropy and mutual information (MI) are used to calculate similarity between miRNAs based on the set of mRNAs interacting with miRNAs.

In events set *X*, information entropy is a measure of the average information content that can be obtained if one of the events actually occurs [38]. This parameter can be defined as

1 $$H(X)=\sum \limits_{x\in X}p(x)\mathit{\log}\frac{1}{p(x)}=-\sum \limits_{x\in X}p(x)\mathit{\log}\left(p(x)\right)$$

where *p*(*x*) is the probability of *x*.

For two discrete random variables *X* and *Y*, their MI can be described as

2 $$I\left(X;Y\right)={\sum}_{x\in X}{\sum}_{y\in Y}p\left(x,y\right)\mathit{\log}\frac{p\left(x,y\right)}{p(x)p(y)}$$

where *p*(*x*) is the marginal probability distribution function of *X*, *p*(*y*) is the marginal probability distribution function of *Y*, and *p*(*x*, *y*) is the joint probability function of *X* and *Y*.

If the mRNAs set of miRNA *A* is${T}_m^A=\left\{{T}_m^A(1),{T}_m^A(2),\dots, {T}_m^A(ma)\right\}$, and the mRNAs set of miRNA *B* is ${T}_m^B=\left\{{T}_m^B(1),{T}_m^B(2),\dots, {T}_m^B(mb)\right\}$ (where *ma* and *mb* are the target genes number of miRNA *A* and miRNA *B*, respectively), then information entropy of ${T}_m^A$ can be calculated as

3 $$\left\{\begin{array}{c}H\left({T}_m^A\right)=-{\sum}_{i=1}^{ma}p\left({T}_m^A(i)\right){\log}_2\left(p\left({T}_m^A(i)\right)\right)\\ {}p\left({T}_m^A(i)\right)=n\left({T}_m^A(i)\right)/N\end{array}\right.$$

where *N* is the total number of the known miRNA–mRNA interactions in the dataset. $n\left({T}_m^A(i)\right)$ is the known number of interactions between the *i*th target gene in the target gene set of miRNA *A* and all miRNAs. $p\left({T}_m^A(i)\right)$ is the rate of the *i*th target gene in the target gene set of miRNA *A* with the known miRNA-mRNA interactions.

The similarity between miRNA *A* and miRNA *B* can use the normalized MI of ${T}_m^A$ and ${T}_m^B$ denoted as

4 $$SM\left(A,B\right)=\frac{2\ast H\left({T}_m^A\cap {T}_m^B\right)}{H\left({T}_m^A\right)+H\left({T}_m^B\right)}$$

where $H\left({T}_m^A\cap {T}_m^B\right)$ is the information entropy of the intersection of ${T}_m^A$ and ${T}_m^B$. When calculating the similarity of miRNA *A* and miRNA *B*, both of their information entropies and the common information entropies of their mRNAs are considered. Also, the frequency of occurrence of the target mRNAs are considered. It measures the similarity between miRNAs by MI according to the occurrence probability of target genes of miRNAs. The target gene with higher probability is more universal and carries less information, while the target gene with lower probability is more specific and carries more information. Obviously, the difference in target gene probability results in such a result. By comparing the similarity data, we find that the metric is determined by the above two factors, and the similarity between the two miRNAs can be appropriately measured.

### Disease similarity network

In building disease similarity network, we first calculate the functional similarity of disease on the basis of disease-gene interaction dataset. We then calculate the semantic similarity of disease on the basis of disease DAG. Finally, we integrate both data into disease similarity to build a disease similarity network.

#### Disease functional similarity of known disease–gene interactions

If the interaction genes set of disease *A* is ${T}_d^A=\left\{{T}_d^A(1),{T}_d^A(2),\dots, {T}_d^A(da)\right\}$, and ${T}_d^B=\left\{{T}_d^B(1),{T}_d^B(2),\dots, {T}_d^B(db)\right\}$ is for disease B (where *da* and *db* are the target genes number of disease *A* and disease *B*, respectively), then the information entropy of ${T}_d^A$ can be calculated as

5 $$\left\{\begin{array}{c}H\left({T}_d^A\right)=-{\sum}_{i=1}^{da}p\left({T}_d^A(i)\right){\log}_2\left(p\left({T}_d^A(i)\right)\right)\\ {}p\left({T}_d^A(i)\right)=n\left({T}_d^A(i)\right)/N\end{array}\right.$$

where *N* is the total number of known disease–gene interactions in the dataset, $n\left({T}_d^A(i)\right)$ is the known number of the interactions between the *i*th target gene in the target gene set of disease *A* and all diseases, and $p\left({T}_d^A(i)\right)$ is the rate of the *i*th target gene in the target gene set of disease *A* with known disease–gene interactions.

The functional similarity between disease *A* and disease *B* can use the normalized MI of ${T}_d^A$ and ${T}_d^B$ denoted as

6 $$SDF\left(A,B\right)=\frac{2\ast H\left({T}_d^A\cap {T}_d^B\right)}{H\left({T}_d^A\right)+H\left({T}_d^B\right)}$$

where $H\left({T}_d^A\right)$ and $H\left({T}_d^B\right)$ are the information entropies ${T}_d^A$ and ${T}_d^B$ of disease *A* and disease *B*, respectively. $H\left({T}_d^A\cap {T}_d^B\right)$ is the information entropy of the intersection of ${T}_d^A$ and ${T}_d^B$. When calculating the functional similarity of disease *A* and disease *B*, both the information entropy of the diseases and the common information entropy of their genes are considered.

#### Disease semantic similarity

Disease semantic similarity *DD* are built from disease DAG as reported in the literature [39].

7 $$DD\left(A,B\right)=\frac{\sum_{t\in {T}_A\cap {T}_B}{D}_A(t)+{\sum}_{t\in {T}_A\cap {T}_B}{D}_B(t)}{2\ast \mathit{\min}\left( DV(A), DV(B)\right)}$$

where *DD*(*A*, *B*) is the semantics similarity value between disease *A* and disease *B* in disease DAG. For the meaning of the symols, please refer to the literature [39].

#### Integrating disease similarity

We integrate disease functional similarity and semantic similarity to obtain disease similarity.

8 $$SD\left(A,B\right)=\gamma \bullet SD F\left(A,B\right)+\left(1-\gamma \right)\bullet DD\left(A,B\right)$$

where *γϵ*(0, 1) is the balance factor to tune the contribution level from disease function similarity and semantic similarity. The results are shown in Additional file 2.

### miRNA similarity network reconstruction

miRNA family information is obtained from miRBase database. We establish the miRNA family information matrix *FAM* for 475 miRNAs in the benchmark dataset. *FAM*(*A*, *B*) = 1 if miRNA *A* and *B* are in the same family; otherwise, *FAM*(*A*, *B*) = 0. We recalculate the miRNA similarity by adding miRNA family information as follows

9 $$miRNAsim\left(A,B\right)= SM\left(A,B\right)\ast \left(1+ FAM\left(A,B\right)\right)$$

We then reconstruct the miRNA similarity network. The results are shown in Additional file 3.

### FCMDAP prediction method

**Fig. 1:**
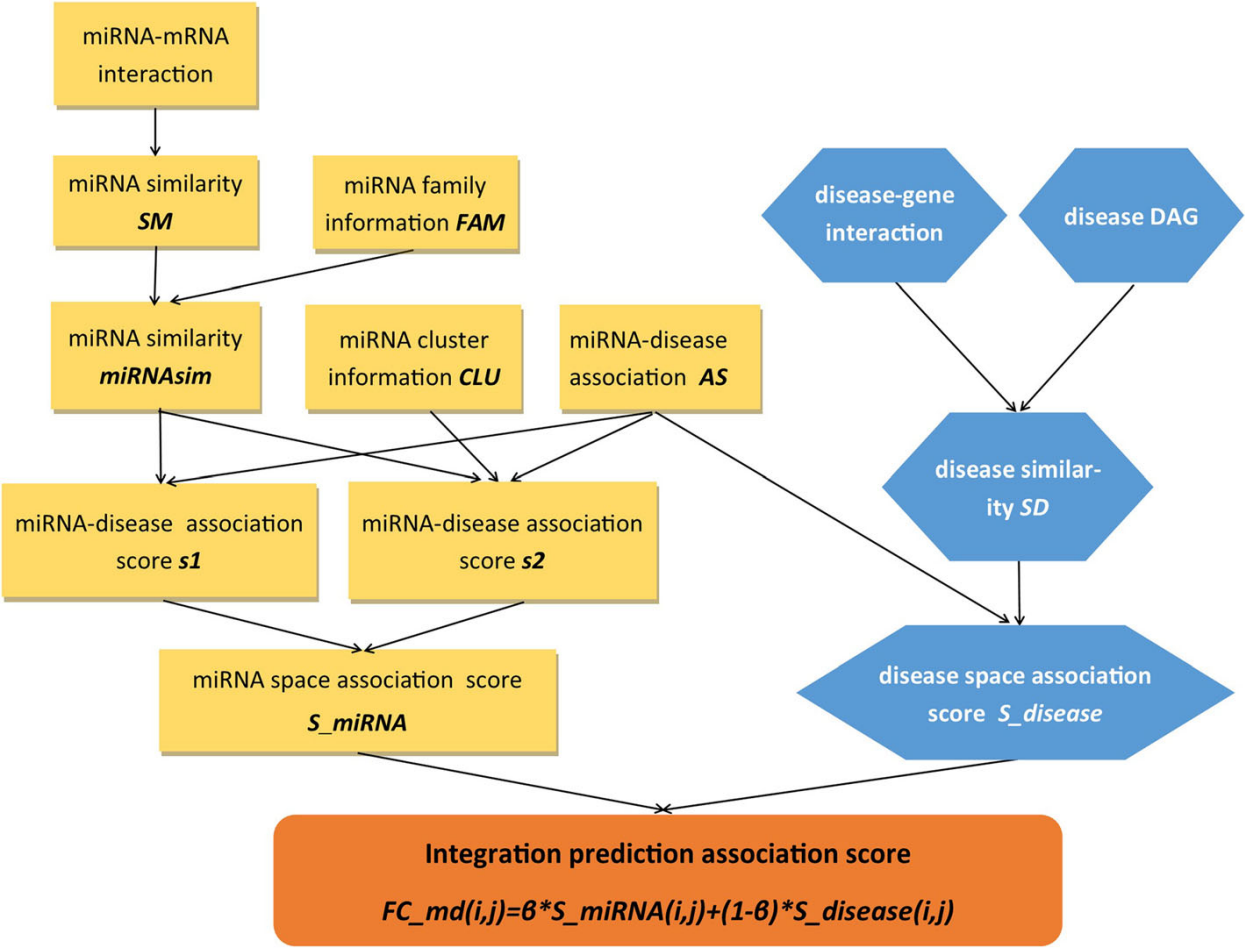
The flowchart of FCMDAP

The flowchart of FCMDAP to predict disease-related miRNAs is shown in Fig. 1.

### miRNA space score calculation

#### Calculating the recommendation score of neighboring miRNAs and disease

Wang et al. [39] proposed that miRNAs with the same similarity tend to be related to diseases with the same functions, and vice versa. In the miRNA space, the related score between miRNA and disease is associated with the correlation score of the neighbor nodes with the miRNA closest to the disease. Hence, if a similar neighbor of a miRNA is related to a disease, then the miRNA may be related to the disease. According to the collaborative recommendation algorithm, the association score of miRNA *i* and disease *j* is calculated based on the similarity scores of the top *k*1 nearest neighbor nodes of miRNA *i* and the association scores of these nodes and disease *j*. We normalize the association score of the top *k*1 most similar neighbor nodes of miRNA *i* and disease *j* by using the following:

10 $$s1\left(i,j\right)=\frac{\sum_{k=1}^{k1} SM1\left(i,k\right)\bullet AS\left(k,j\right)}{\sum \limits_{k=1}^{k1} SM1\left(i,k\right)}$$

where *SM*1 is the row vector of each miRNA in the miRNA matrix *miRNAsim* and is sorted in descending order. Hence, miRNAs that are more similar will be ranked higher. *SM*1(*i*, *k*) is a component of miRNA *i* and the *kth* closet similar neighbor nodes in the vector *SM*1. If miRNA *k* is related to disease *j*, then we calculate the sum of the related scores between miRNA *i* and miRNA *k* and divide the sum of the related scores of the top *k*1 similar neighbor nodes of miRNA *i*.

#### Calculating the prediction score in the same miRNA cluster

Baskerville S. and Bartel D.P. [40] found significant coexpression among the proximal pairs of miRNAs (< 50 kb). The closest miRNA cluster is usually expressed as a common regulatory unit of polycistronics, and intronic miRNAs are usually coexpressed with host genes, presenting complex miRNA expression patterns. Lu et al. [41] performed statistical analysis and found that miRNAs in 46% of diseases have at least one neighboring member. For example, all of the 6 miRNAs (miR-17, miR-18a, miR-19a, miR-20a, miR-19b-1 and miR-92a-1) involved in hematopoietic malignancies are located in the miR-17 cluster. This result shows that neighboring miRNAs may be regulated by a common regulator under the same conditions and interactions, and their dysfunction may lead to the same disease. Wang et al. [39] confirmed that miRNAs are more likely to associate with the similar disease when clustered and located within 20 kb of genomic location. We downloaded the information of the location of human miRNAs in the genome from miRBase v.21, and clustered miRNAs are selected within a distance of 20 kb. A miRNA cluster matrix *CLU* is built for the 475 miRNAs in the benchmark dataset. Basing on the collaborative recommendation algorithm, we calculate the normalized related scores between miRNA *i* and disease *j* as

11 $$s2\left(i,j\right)=\frac{\sum_{k=1}^n SM2\left(i,k\right)\bullet AS\left(k,j\right)}{\sum \limits_{k=1}^n SM2\left(i,k\right)}$$

where *SM*2(*i*, *k*) is the similarity score of miRNA *i* and miRNA *k* in the same cluster, and *n* is the number of miRNAs in the same cluster as miRNA *i*. If miRNA *k* is related to disease *j*, then we add the similarity score *miRNAsim*(*i*, *k*) of miRNA *i* and miRNA *k* and divide the sum of the similarity score of pairwise miRNAs in the same cluster as miRNA *i*. From the formula, we can find that the closer the miRNAs are in the same cluster with disease *j*, the closer the relation of miRNA *i* with disease *j* will be.

#### Integrating similarity score in miRNA space

In the miRNA space, the recommendation scores of miRNA–disease associations are calculated by integrating the score of top *k* similarity neighboring miRNAs of miRNA *i* and the recommendation score of miRNAs in the same cluster as miRNA *i* with disease *j*. The formula is as follows:

12 $$S\_ miRNA\left(i,j\right)=\alpha \ast s1\left(i,j\right)+\left(1-\alpha \right)\ast s2\left(i,j\right)$$

where *α* is a tradeoff factor. Experiments show that FCMDAP gets the best performance when *α* is 0.5.

### Calculating disease space score

In the disease space, we also use the *k*-nearest neighbor-based recommendation algorithm to calculate the predicted association score between disease and miRNA. If the *k*-nearest neighbor of a disease is related to a miRNA, then the disease is related to the miRNA.

According to the collaborative recommendation algorithm, for miRNA *i* with disease *j*, their recommendation score is calculated by the normalized similarity score between the *k*2-nearest neighbors of disease *j* and miRNA *i*. The formula is shown as follows

13 $$S\_ disease\left(i,j\right)=\frac{\sum_{k=1}^{k2} AS\left(i,k\right)\bullet SD1\left(k,j\right)}{\sum \limits_{k=1}^{k2} SD1\left(k,j\right)}$$

where *SD*1 is the column vector of all diseases in disease similarity matrix *SD*. These vectors are sorted in descending order, and the most similar disease is ranked as the highest. *SD*1(*k*, *j*) represents the *k*-th component of the *k*-th nearest neighbor of disease *j* on the similarity column vector *SD* of disease *j*.

### Calculating the final prediction score of disease-related miRNAs

The final prediction score of disease-related miRNAs of miRNA *i* with disease *j* is obtained by integrating the scores in miRNA space and disease space as follows

14 $$FC\_ md\left(i,j\right)=\beta \ast S\_ mi\mathrm{R} NA\left(i,j\right)+\left(1-\beta \right)\ast S\_ disease\left(i,j\right)$$

where *β* is the factor used to balance the weight of two spaces. Experiments show that the optimal performance of FCMDAP can be obtained when the value of β is 0.8.

FCMDAP can predict isolated disease-related miRNAs and isolated miRNA-related diseases. Isolated disease-related miRNAs/miRNA-related diseases are miRNAs/diseases without any related diseases/miRNAs, such as newly discovered miRNAs/diseases. When we use FCMDAP to predict isolated disease-related miRNAs, all miRNAs related to disease *j* do not exist, leading to the prediction score *S* _ *miRNA*(*i*, *j*) of 0. We calculate *S* _ *disease*(*i*, *j*) from two parts, namely, similarity score between miRNA *i* and other diseases and similarity between diseases. Thus, FCMDAP can predict the association between isolated diseases and miRNAs. When we predict isolated miRNA-related disease, diseases related to miRNA *i* do not exist, leading *S* _ *disease*(*i*, *j*)= 0. We can calculate *S* _ *miRNA*(*i*, *j*) from the relationship between other miRNA and disease *j* and the similarity between miRNAs to predict the association of miRNA *i* and disease *j*.

## Results

### Characteristics of the miRNA-disease association network

The benchmark data set include 5048 known miRNA–disease associations of 475 miRNAs and 334 diseases. The characteristics of these associations are shown in Table 1. The average degree of diseases and miRNAs are 15.11 and 10.63, respectively.

**Table 1:** Global characteristic of the known miRNA-disease association network

Characteristic	Number
No. of miRNA-disease association	5048
No. of miRNAs	475
No. of diseases	334
Avg. degree of miRNAs	10.63
Avg. degree of diseases	15.11
Max degree of miRNAs	112
Min degree of miRNAs	1
Max degree of diseases	208
Min degree of diseases	1

### Performance evaluation of FCMDAP

The LOOCV of known miRNA-disease associations is used to evaluate the performance of FCMDAP. For a given disease *d*, each known association of disease *d* is deleted in turn as a test sample, and the other known associations are used as training set. The remaining miRNAs without experimental evidence regarding their relation with disease *d* comprise the candidate miRNA set. The association prediction scores of these candidate miRNAs and diseases are calculated and ranked. If the rank exceeds a given threshold, then we consider FCMDAP to successfully predict the association of miRNA and disease. After changing the threshold, drawing the receiver operating characteristic (ROC) curve and calculating the area under the curve (AUC) value are conducted to evaluate prediction performance.

The ROC plots indicate the relationship between the true positive rate (TPR) and the false positive rate (FPR) at different thresholds. If TP, FP, TN, and FN represent true positive, false positive, true negative, and false negative, respectively, then TPR and FPR are calculated as

15 $$TPR=\frac{TP}{TP+ FN}$$

and

16 $$FPR=\frac{FP}{TN+ FP}$$

After one round of LOOCV, one association between miRNA and disease was excluded, and the prediction score was calculated by remaining associations. All these scores were sorted and a special ranking position was selected as threshold. TP and FP are the number of experimentally verified and unverified associations above the threshold, respectively. TN and FN are the number of unverified and verified associationas below the threshold, respectively.

**Fig. 2:**
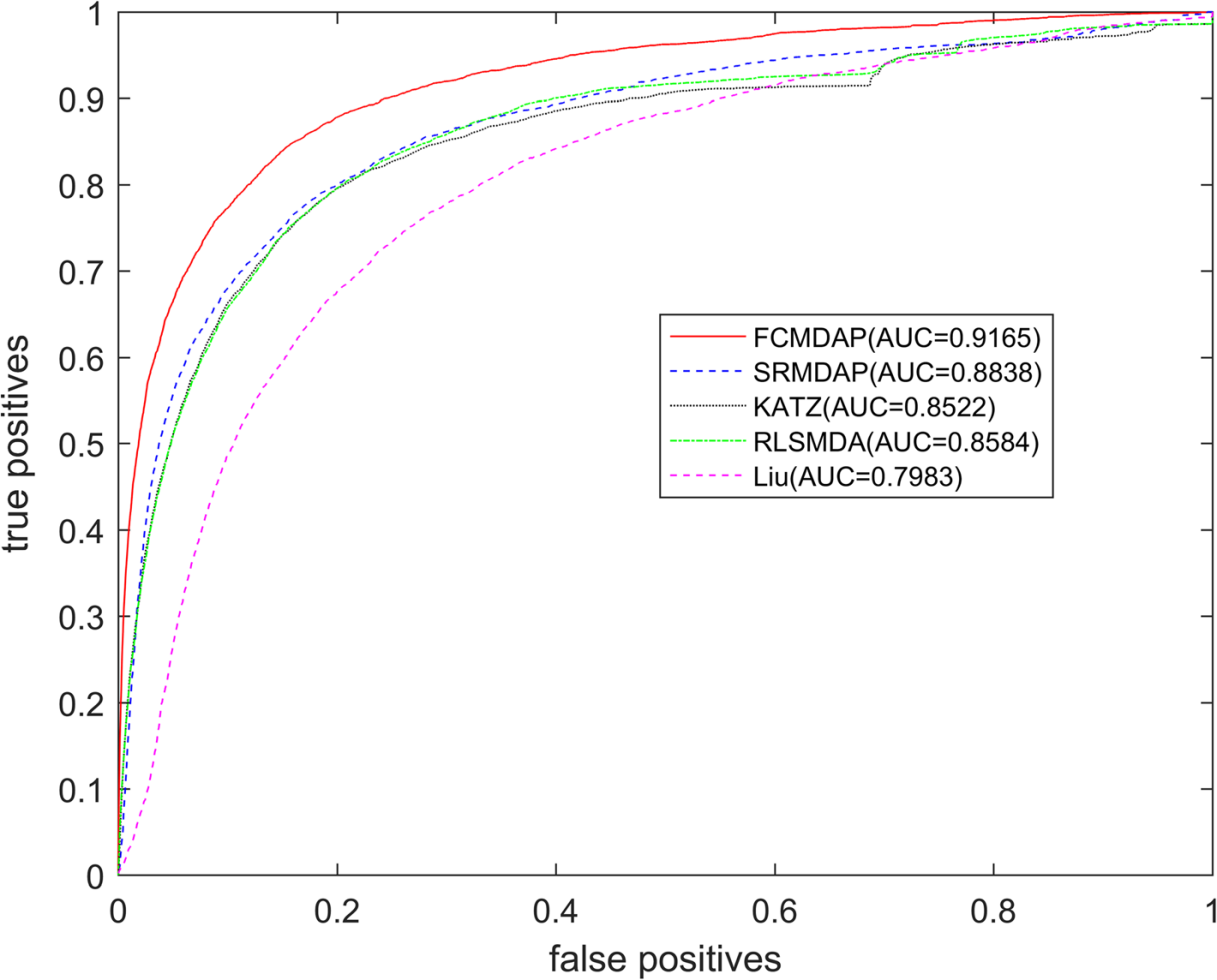
The ROC curve and AUC value of FCMDAP and other compared methods

We compared FCMDAP with SRMDAP, RLSMDA [28], KATZ [23], and Liu’s method [31] in terms of prediction performance, AUC value, and ROC shapes on the benchmark data set. The values of the four parameters of FCMDAP are *α* = 0.5, *β* = 0.8, *k*1 = 50, and *k*2 = 30. The optimal parameters of SRMDAP, RLSMDA, KATZ, and Liu’s method are set as previously described. The comparison of the overall ROC curves and AUCs of all methods are shown in Fig. 2. The average AUC value of FCMDAP is 0.9165, which is 3.72, 5.81, 6.43, and 11.82% higher than those of SRMDAP, RLSMDA, KATZ and Liu’s method, respecitively. When the FPR is lower than 0.2, the ROC of FCMDAP is more convex near the upper left corner, indicating that the prediction accuracy is higher. Therefore, FCMDAP shows higher prediction accuracy than the other methods.

To obtain reliable judgment, we tested 18 human diseases associated with at least 70 miRNAs. The results are shown in Table 2. Table 2 shows that FCMDAP obtained the highest AUC value of 0.8837 for pancreatic neoplasms and the lowest AUC value of 0.7572 for hepatocellular carcinoma. The average AUC value for the 18 diseases is 0.8195. The average AUC values for the 18 diseases obtained from SRMDAP, RLAMDA, KATA, and Liu’s method are 0.8057, 0.6671, 0.6901, and 0.5178, respectively. The average AUC value obtained by FCMDAP is 1.38, 15.24, 12.94, and 30.17% higher than those of the four methods, respectively. Hence, FCMDAP exhibits better performance than SRMAPS, RLSMDA, KATA, and Liu’s method.

**Table 2:** AUC value of compared five methods for 18 diseases

Disease names	No. of related miRNAs	FCMDAP	SRMDAP	RLSMDA	KATZ	Liu’s method
Carcinoma, Hepatocellular	208	0.7572	0.7639	0.6909	0.6881	0.4807
Breast Neoplasms	197	0.7733	0.7776	0.6814	0.6779	0.4147
Stomach Neoplasms	174	0.7658	0.7591	0.6635	0.6791	0.5498
Colorectal Neoplasms	143	0.7904	0.7929	0.6647	0.6895	0.4699
Melanoma	136	0.8300	0.7958	0.6584	0.6673	0.4804
Lung Neoplasms	128	0.8688	0.8874	0.7198	0.7675	0.5243
Heart Failure	120	0.7737	0.7538	0.6608	0.6622	0.5040
Prostatic Neoplasms	116	0.8185	0.8076	0.6704	0.7054	0.5440
Ovarian Neoplasms	112	0.8684	0.8732	0.7194	0.7705	0.5382
Carcinoma, Renal Cell	104	0.7878	0.7367	0.5815	0.6126	0.4932
Pancreatic Neoplasms	97	0.8837	0.8687	0.6829	0.7288	0.5355
Carcinoma, Non-Small-Cell Lung	94	0.8417	0.8322	0.6873	0.6981	0.5470
Glioblastoma	94	0.8383	0.7686	0.6421	0.6522	0.5644
Urinary Bladder Neoplasms	90	0.8214	0.7935	0.6231	0.6635	0.5475
Carcinoma, Squamous Cell	78	0.8640	0.8637	0.7179	0.7200	0.5398
Colonic Neoplasms	77	0.8278	0.8271	0.6582	0.6859	0.5490
Glioma	71	0.8679	0.8212	0.6727	0.7146	0.5591
Esophageal Neoplasms	70	0.7723	0.7789	0.6126	0.6383	0.4781
Average AUC value		0.8195	0.8057	0.6671	0.6901	0.5178

### Parameter effect

**Fig. 3:**
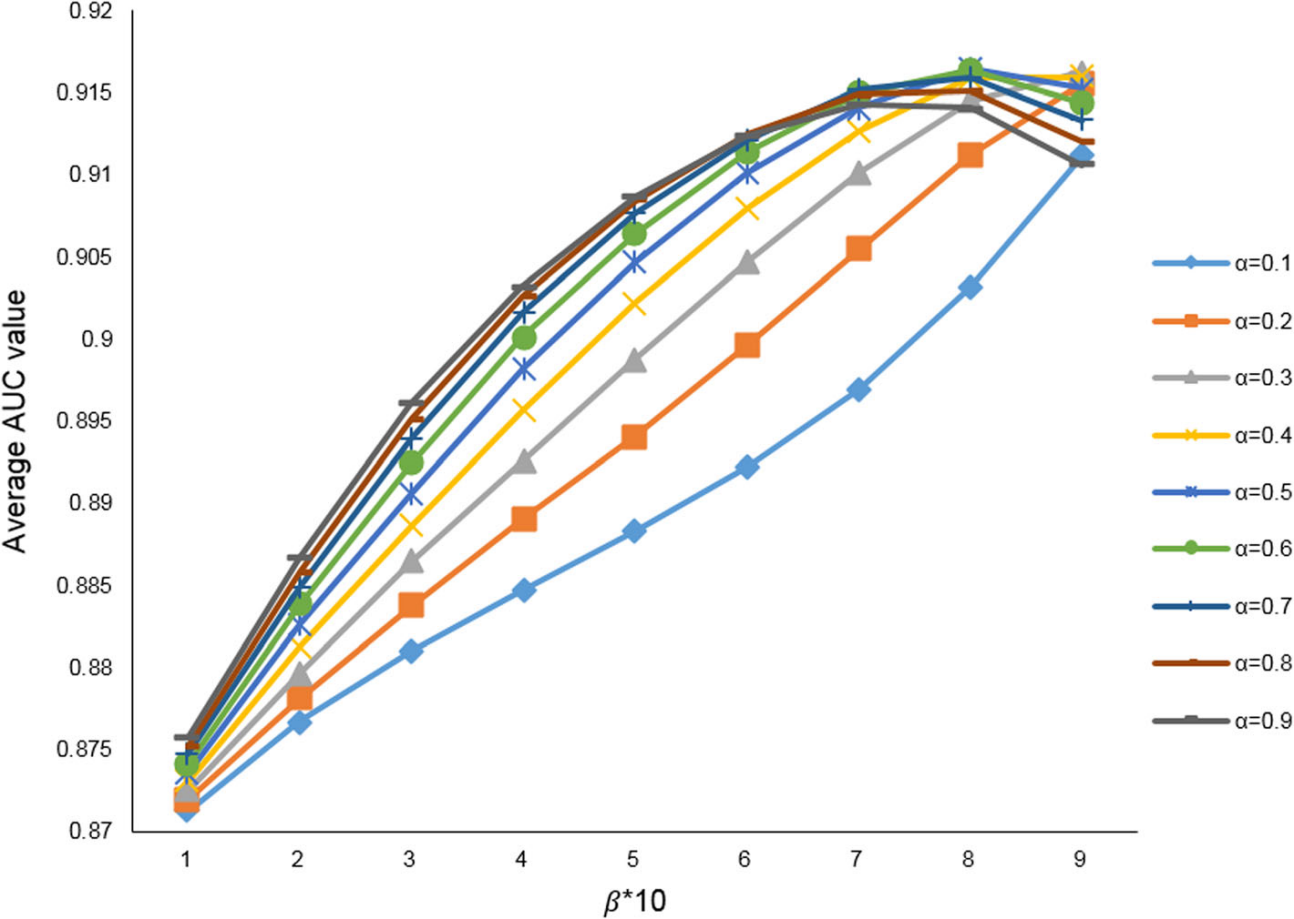
Average AUCs value affected by *α*, *β*

The five parameters in FCMDAP are *α*, *β*, *γ*, *k*1, and *k*2. We focus on miRNA space. In the miRNA space, *α* balances the tradeoff between the recommendation score from the neighboring miRNAs and the score from the miRNA cluster. *β* is the entire space balancing factor that sets different weights of recommendation scores from the miRNA and disease spaces. To obtain optimal parameters, we assign different values to *α* and *β* starting from 0.1 to calculate the recommendation scores of miRNA–disease association and evaluate the performance of FCMDAP by calculating AUC value. We repeat this work by increasing *α* and *β* in steps of 0.1 and calculating the AUC value until *α* and *β* are both 1. We obtain the best performance when *α* = 0.5 and *β* = 0.8, and the AUC of FCMDAP is 0.9165. The results are shown in Fig. 3.

As shown in Fig. 3, the ordinate is the average AUC value, and the abscissa is the value at which *β* is magnified 10 times. Each curve in the figure represents the line connecting the points of the corresponding average AUC values when the same *α* value differs from the *β* value. The average AUC value varies from 0.8712 to 0.9165. When *α* = 0.1, *β* = 0.1, the average AUC is the minimum value of 0.8712. When *α* = 0.5, *β* = 0.8, the average AUC is the maximum value of 0.9165. The general trend is that the overall average AUC value increase with increasing *α*, *β*. *γ* denotes the balance factor in the disease similarity network based on disease functional similarity in disease–gene interactions and disease semantic similarity in disease DAG. *k*1 and *k*2 denotes the number of neighboring miRNAs and neighboring diseases in the recommendation algorithm, respectively. The values of *γ*, *k*1, and *k*2 are set as 0.5, 50, and 30, respectively, according to experience.

### Case studies

Three important diseases (colorectal neoplasms, lung neoplasms, and pancreatic neoplasms) were selected to evaluate the performance of FCMDAP. The top 50 miRNA candidates of these three diseases were analyzed and verified using miRCancer (v. Oct. 2017), dbDEMC (v. 2.0), and PhenomiR (v. 2.0) databases and findings in the literature.

Colorectal neoplasms, the third most common cancer worldwide, severely affects the human health. In this regard, understanding colorectal-related miRNAs is important for diagnosis and prognosis of colorectal neoplasmsa. For example, patients with early colorectal neoplasms can be discriminated from healthy people by using serum miR-21, miR-29a, and miR-125b levels [42]. We used experimentally identified miRNA–disease associations as training samples to calculate the recommendation score of all candidate miRNAs through FCMDAP. We then ranked them in descending order and selected the top 50 miRNAs for verification. The top 50 candidate miRNAs and the corresponding evidence of their association with colorectal neoplasms are listed in Table 3. All the top 50 miRNAs were confirmed by analysis of miRCancer, dbDEMC, and PhenomiR databases.

**Table 3:** The top 50 candidate miRNAs associated with colorectal neoplasms predicted by FCMDAP and the confirmation for their associations by miRCancer, PhenomiR or dbDEMC databases are listed here. All of them have been confirmed

Rank	miRNA	Evidence
1	hsa-mir-106b	miRCancer,dbDEMC,PhenomiR
2	hsa-mir-29b	miRCancer,dbDEMC,PhenomiR
3	hsa-mir-15a	miRCancer,dbDEMC,PhenomiR
4	hsa-mir-100	miRCancer,dbDEMC,PhenomiR
5	hsa-mir-192	miRCancer,dbDEMC,PhenomiR
6	hsa-mir-208b	dbDEMC
7	hsa-mir-24	miRCancer,dbDEMC,PhenomiR
8	hsa-let-7f	dbDEMC,PhenomiR
9	hsa-mir-101	miRCancer,dbDEMC,PhenomiR
10	hsa-let-7 g	dbDEMC,PhenomiR
11	hsa-mir-15b	miRCancer,dbDEMC,PhenomiR
12	hsa-mir-20b	dbDEMC
13	hsa-mir-193b	miRCancer,dbDEMC
14	hsa-mir-615	dbDEMC
15	hsa-mir-30c	dbDEMC,PhenomiR
16	hsa-mir-223	miRCancer,dbDEMC,PhenomiR
17	hsa-mir-130b	miRCancer,dbDEMC,PhenomiR
18	hsa-mir-296	miRCancer,dbDEMC,PhenomiR
19	hsa-mir-98	dbDEMC,PhenomiR
20	hsa-mir-125a	miRCancer,dbDEMC,PhenomiR
21	hsa-mir-29c	dbDEMC,PhenomiR
22	hsa-let-7d	dbDEMC,PhenomiR
23	hsa-mir-205	miRCancer,dbDEMC,PhenomiR
24	hsa-mir-23b	miRCancer,dbDEMC,PhenomiR
25	hsa-mir-10a	miRCancer,dbDEMC,PhenomiR
26	hsa-mir-128	miRCancer,dbDEMC,PhenomiR
27	hsa-mir-744	dbDEMC
28	hsa-mir-484	dbDEMC,PhenomiR
29	hsa-mir-32	miRCancer,dbDEMC,PhenomiR
30	hsa-mir-197	dbDEMC,PhenomiR
31	hsa-mir-151a	dbDEMC
32	hsa-mir-331	miRCancer,dbDEMC,PhenomiR
33	hsa-mir-138	miRCancer,dbDEMC,PhenomiR
34	hsa-mir-181d	dbDEMC
35	hsa-mir-449a	miRCancer,PhenomiR
36	hsa-mir-449c	dbDEMC
37	hsa-mir-326	miRCancer,dbDEMC,PhenomiR
38	hsa-mir-212	miRCancer,dbDEMC,PhenomiR
39	hsa-mir-196b	miRCancer,dbDEMC
40	hsa-mir-191	miRCancer,dbDEMC,PhenomiR
41	hsa-mir-30d	dbDEMC,PhenomiR
42	hsa-mir-214	miRCancer,dbDEMC,PhenomiR
43	hsa-mir-204	miRCancer,dbDEMC,PhenomiR
44	hsa-mir-99b	dbDEMC,PhenomiR
45	hsa-mir-449b	dbDEMC
46	hsa-mir-769	dbDEMC
47	hsa-mir-520 h	dbDEMC
48	hsa-mir-181c	dbDEMC,PhenomiR
49	hsa-mir-520 g	dbDEMC
50	hsa-mir-361	miRCancer,dbDEMC

Lung neoplasms is a malignant lung tumor caused by uncontrolled growth of lung tissue cells. Lung tumor cells can also rapidly spread from the lungs to other nearby tissues or other parts of the body. According to the World Health Organization’s 2014 World Cancer Report [43], the number of patients with lung tumors worldwide reached 1.8 million in 2012. Lung neoplasms are the main cause of cancer-related death in men and women (other than breast neoplasms). In the United States, the 5-year survival rate for patients diagnosed with lung neoplasms is only 17.4%, which is lower than that in developing countries. Thus, effective methods for early diagnosis and treatment of lung neoplasms are important. Evidence indicates the important role of miRNAs in the pathogenesis, migration, and spread of lung neoplasms. For example, Takamizawa et al. [3] first found that the expression levels of let-7 are often reduced in lung neoplasms in vitro and in vivo in their study on 143 cases of lung neoplasms. The decrease in let-7 expression may affect the survival of patients that with lung neoplasms who were surgically treated. Johnson et al. [44] found that let-7 acts as a tumor suppressor in lung cells and negatively regulates the expression of the oncogene RAS. Hence, miRNAs can be used to develop drugs for treatment of lung tumors.

In our work, we used experimentally identified miRNA–disease associations as training samples to calculate recommendation scores of all candidate miRNAs based on FCMDAP. We then ranked them in descending order and selected the top 50 miRNAs for verification. The top 50 candidate miRNAs and the corresponding evidence of their association with colorectal neoplasms are listed in Table 4. Among these miRNAs, 48 miRNAs were confirmed in miRCancer, dbDEMC, and PhenomiR databases, and only two miRNAs (hsa-mir-520 g, hsa-mir-147a) were not confirmed. A recent study (PMID: 29033588) [45] showed that hsa-mir-147a is related to lung neoplasms. In this study, lncRNA HOXD-AS1 is specifically upregulated in non-small-cell lung cancer (NSCLC) tissues and promotes cancer cell growth by targeting miR-147a.

**Table 4:** The top 50 candidate miRNAs associated with lung neoplasms predicted by FCMDAP and the confirmation for their associations by miRCancer, PhenomiR or dbDEMC databases are listed here. 49 of them have been confirmed

Rank	miRNA	Evidence
1	hsa-mir-429	dbDEMC,miRCancer
2	hsa-mir-141	dbDEMC,PhenomiR,miRCancer
3	hsa-mir-106b	dbDEMC,PhenomiR
4	hsa-mir-520 g	unconfirmed
5	hsa-mir-16	dbDEMC,PhenomiR,miRCancer
6	hsa-mir-215	dbDEMC,PhenomiR
7	hsa-mir-217	dbDEMC,PhenomiR,miRCancer
8	hsa-mir-376c	dbDEMC,PhenomiR
9	hsa-mir-181d	dbDEMC,PhenomiR
10	hsa-mir-20b	dbDEMC,PhenomiR
11	hsa-mir-15a	dbDEMC,PhenomiR,miRCancer
12	hsa-mir-195	dbDEMC,PhenomiR,miRCancer
13	hsa-mir-451a	dbDEMC
14	hsa-mir-99a	dbDEMC,PhenomiR
15	hsa-mir-193b	dbDEMC,PhenomiR
16	hsa-mir-130b	dbDEMC,PhenomiR,miRCancer
17	hsa-mir-194	dbDEMC,PhenomiR,miRCancer
18	hsa-mir-130a	dbDEMC,PhenomiR
19	hsa-mir-373	dbDEMC,PhenomiR
20	hsa-mir-15b	dbDEMC,PhenomiR,miRCancer
21	hsa-mir-10a	dbDEMC,PhenomiR
22	hsa-mir-378a	dbDEMC
23	hsa-mir-122	dbDEMC,PhenomiR
24	hsa-mir-449a	dbDEMC,PhenomiR,miRCancer
25	hsa-mir-148b	dbDEMC,PhenomiR,miRCancer
26	hsa-mir-449b	dbDEMC,PhenomiR,miRCancer
27	hsa-mir-204	dbDEMC,PhenomiR
28	hsa-mir-615	dbDEMC
29	hsa-mir-383	dbDEMC,PhenomiR,miRCancer
30	hsa-mir-340	dbDEMC,PhenomiR
31	hsa-mir-328	dbDEMC,PhenomiR
32	hsa-mir-151a	dbDEMC
33	hsa-mir-152	dbDEMC,PhenomiR
34	hsa-mir-153	dbDEMC,PhenomiR,miRCancer
35	hsa-mir-320a	dbDEMC,PhenomiR
36	hsa-mir-302d	dbDEMC,PhenomiR
37	hsa-mir-630	dbDEMC,miRCancer
38	hsa-mir-296	dbDEMC,PhenomiR
39	hsa-mir-139	dbDEMC,PhenomiR
40	hsa-mir-149	dbDEMC,PhenomiR
41	hsa-mir-423	dbDEMC,PhenomiR
42	hsa-mir-23b	dbDEMC,PhenomiR
43	hsa-mir-196b	dbDEMC,PhenomiR
44	hsa-mir-147a	PMID:29144017
45	hsa-mir-425	dbDEMC,PhenomiR
46	hsa-mir-99b	dbDEMC,PhenomiR,miRCancer
47	hsa-mir-324	dbDEMC,PhenomiR
48	hsa-mir-302c	dbDEMC,PhenomiR
49	hsa-mir-421	dbDEMC,PhenomiR
50	hsa-mir-484	dbDEMC,PhenomiR

Pancreatic neoplasms are cellular masses caused by uncontrollable pancreatic cell proliferation. The most common symptoms of pancreatic neoplasms include yellowing of the skin, abdominal or back pain, unexplained weight loss, and loss of appetite. Early pancreatic neoplasms are small and have no symptoms. Most pancreatic neoplasms are large when they are found and can metastasize to other parts of the body. According to reports, 411,600 people worldwide died of various pancreatic neoplasms in 2015. Pancreatic neoplasms most often occur in developed countries; that is, these malignancies rank as the fifth most common cancer in the UK and the fourth most common cancer in the United States [43, 46]. The prognosis of pancreatic neoplasms is very poor, with 25% survival rate for 1 year after diagnosis and 5% survival rate for 5 years. Thus, effective methods for early diagnosis, treatment, and prognosis of pancreatic neoplasms must be developed. At present, evidence supports the role of miRNA differential expression in the diagnosis, treatment, and prognosis of pancreatic neoplasms. For example, Sadakari et al. [47] found that the relative expression levels of miR-21 and miR-155 in tissues and pancreatic juice of patients with pancreatic ductal adenocarcinoma are significantly higher than those in patients with chronic pancreatitis; thus, miR-21 and miR-155 in pancreatic juice may be a potential biomarker for diagnosis of pancreatic ductal adenocarcinoma. Lodygin et al. [48] reported that the expression of miR-34a is silenced in several types of cancers, including pancreatic neoplasms, due to CpG methylation. By partially targeting CDK16, the re-expression of miR-34a in MiaPaC2 cell line with pancreatic neoplasms induces cellular senescence and cell cycle arrest. This observation indicates that miR-34a is a neoplasm suppressor gene, which is inactivated by CpG methylation and subsequent transcriptional silencing in various tumors, such as pancreatic neoplasms. Thus, miR-34a can be used as a therapeutic target for malignant neoplasms, such as pancreatic neoplasms.

In our work, we also calculated the recommendation score of all candidate miRNAs based on FCMDAP, ranked them in descending order, and selected the top 50 miRNAs for verification. The top 50 candidate miRNAs and the corresponding evidence of their associations with pancreatic neoplasms are listed in Table 5. Among the top 50 miRNAs, 48 miRNAs were confirmed in the miRCancer, dbDEMC, and PhenomiR databases, and only two miRNAs (miR-378a and miR-365a) were not confirmed.

**Table 5:** The top 50 candidate miRNAs associated with pancreatic neoplasms predicted by FCMDAP and the confirmation for their associations by miRCancer, PhenomiR or dbDEMC databases are listed here. 48 of them have been confirmed

Rank	miRNA	Evidence
1	hsa-mir-141	dbDEMC,PhenomiR,miRCancer
2	hsa-mir-29a	dbDEMC,PhenomiR,miRCancer
3	hsa-mir-181a	dbDEMC,PhenomiR,miRCancer
4	hsa-mir-29c	dbDEMC,PhenomiR,miRCancer
5	hsa-mir-19b	dbDEMC,PhenomiR
6	hsa-mir-93	dbDEMC,PhenomiR
7	hsa-mir-30a	dbDEMC,PhenomiR
8	hsa-mir-1	dbDEMC,PhenomiR
9	hsa-mir-98	dbDEMC,PhenomiR
10	hsa-mir-106b	dbDEMC,PhenomiR
11	hsa-mir-215	dbDEMC,PhenomiR,miRCancer
12	hsa-mir-520 g	dbDEMC
13	hsa-mir-7	dbDEMC,PhenomiR,miRCancer
14	hsa-mir-9	dbDEMC,PhenomiR
15	hsa-mir-195	dbDEMC,PhenomiR
16	hsa-mir-19a	dbDEMC,PhenomiR
17	hsa-mir-181d	dbDEMC,PhenomiR
18	hsa-mir-193b	dbDEMC
19	hsa-mir-125a	dbDEMC,PhenomiR
20	hsa-mir-135a	dbDEMC,PhenomiR
21	hsa-mir-205	PhenomiR,miRCancer
22	hsa-mir-26b	dbDEMC,PhenomiR
23	hsa-mir-138	dbDEMC,PhenomiR,miRCancer
24	hsa-mir-181c	dbDEMC,PhenomiR,miRCancer
25	hsa-mir-136	dbDEMC,PhenomiR
26	hsa-mir-133a	dbDEMC,PhenomiR,miRCancer
27	hsa-mir-320a	dbDEMC,PhenomiR,miRCancer
28	hsa-mir-20b	dbDEMC
29	hsa-mir-449a	dbDEMC
30	hsa-mir-615	dbDEMC,miRCancer
31	hsa-mir-140	dbDEMC,PhenomiR
32	hsa-mir-335	dbDEMC,PhenomiR,miRCancer
33	hsa-mir-378a	unconfirmed
34	hsa-mir-130b	dbDEMC,PhenomiR
35	hsa-mir-365a	unconfirmed
36	hsa-mir-423	dbDEMC,PhenomiR
37	hsa-mir-23b	dbDEMC,PhenomiR
38	hsa-mir-373	dbDEMC,PhenomiR,miRCancer
39	hsa-mir-149	dbDEMC,PhenomiR
40	hsa-mir-153	dbDEMC,PhenomiR
41	hsa-mir-30b	dbDEMC,PhenomiR
42	hsa-mir-27b	dbDEMC,PhenomiR
43	hsa-mir-22	dbDEMC,PhenomiR
44	hsa-mir-324	dbDEMC,PhenomiR
45	hsa-mir-185	dbDEMC,PhenomiR
46	hsa-mir-744	dbDEMC,miRCancer
47	hsa-mir-484	dbDEMC
48	hsa-mir-449b	dbDEMC
49	hsa-mir-328	dbDEMC,PhenomiR
50	hsa-mir-148b	dbDEMC,PhenomiR,miRCancer

### Predicting isolated diseases and isolated miRNAs

FCMDAP can predict isolated disease-related miRNAs. In our work, we removed all experimentally verified disease-miRNA associations for a given disease and calculated the recommendation score by FCMDAP. We also ranked the miRNAs according to their recommendation scores. The average AUC of FCMDAP for predicting an isolated disease is 0.8417. For lung neoplasms, FCMDAP identifies the top 50 miRNAs related to lung neoplasms (Table 6). All of the top 50 miRNAs were confirmed by one or more databases (miRCancer, dbDEMC, or PhenomiR). Hence, FCMDAP exhibits satisfactory performance in predicting isolated diseases.

**Table 6:** The top 50 miRNAs associated with isolated lung neoplasms predicted by FCMDAP and their evidence

Rank	miRNA	Evidence
1	hsa-mir-16	dbDEMC, PhenomiR, miRCancer
2	hsa-mir-15a	dbDEMC, PhenomiR, miRCancer
3	hsa-mir-195	dbDEMC, PhenomiR, miRCancer
4	hsa-mir-141	dbDEMC, PhenomiR, miRCancer
5	hsa-mir-106b	dbDEMC, PhenomiR
6	hsa-mir-429	dbDEMC, miRCancer
7	hsa-mir-296	dbDEMC, PhenomiR
8	hsa-mir-151a	dbDEMC
9	hsa-mir-122	dbDEMC, PhenomiR
10	hsa-mir-451a	dbDEMC
11	hsa-mir-130a	dbDEMC, PhenomiR
12	hsa-mir-378a	dbDEMC
13	hsa-mir-99a	dbDEMC, PhenomiR
14	hsa-mir-302b	dbDEMC, PhenomiR, miRCancer
15	hsa-mir-152	dbDEMC, PhenomiR
16	hsa-mir-193b	dbDEMC, PhenomiR
17	hsa-mir-708	dbDEMC
18	hsa-mir-625	dbDEMC
19	hsa-mir-204	dbDEMC, PhenomiR
20	hsa-mir-15b	dbDEMC, PhenomiR, miRCancer
21	hsa-mir-302c	dbDEMC, PhenomiR
22	hsa-mir-194	dbDEMC, PhenomiR, miRCancer
23	hsa-mir-320a	dbDEMC, PhenomiR
24	hsa-mir-449a	dbDEMC, PhenomiR, miRCancer
25	hsa-mir-149	dbDEMC, PhenomiR
26	hsa-mir-129	dbDEMC, PhenomiR, miRCancer
27	hsa-mir-20b	dbDEMC, PhenomiR
28	hsa-mir-139	dbDEMC, PhenomiR
29	hsa-mir-302a	dbDEMC, PhenomiR
30	hsa-mir-148b	dbDEMC, PhenomiR, miRCancer
31	hsa-mir-10a	dbDEMC, PhenomiR
32	hsa-mir-328	dbDEMC, PhenomiR
33	hsa-mir-215	dbDEMC, PhenomiR
34	hsa-mir-99b	dbDEMC, PhenomiR, miRCancer
35	hsa-mir-302d	dbDEMC, PhenomiR
36	hsa-mir-196b	dbDEMC, PhenomiR
37	hsa-mir-151b	dbDEMC
38	hsa-mir-373	dbDEMC, PhenomiR
39	hsa-mir-345	dbDEMC, PhenomiR
40	hsa-mir-449b	dbDEMC, PhenomiR, miRCancer
41	hsa-mir-452	dbDEMC, PhenomiR
42	hsa-mir-339	dbDEMC, PhenomiR
43	hsa-mir-367	dbDEMC, PhenomiR
44	hsa-mir-342	dbDEMC, PhenomiR
45	hsa-mir-130b	dbDEMC, PhenomiR, miRCancer
46	hsa-mir-211	dbDEMC, PhenomiR
47	hsa-mir-92b	dbDEMC, PhenomiR
48	hsa-mir-520c	dbDEMC
49	hsa-mir-520d	dbDEMC
50	hsa-mir-520a	dbDEMC

FCMDAP also shows satisfactory performance in predicting isolated miRNA-related diseases. In our work, we removed all disease association information for a given miRNA and calculated the recommendation score for all diseases for a given miRNA by using FCMDAP. We ranked these diseases and verified them in the databases. The average AUC of the FCMDAP to predict isolated miRNA is 0.8944. For hsa-mir-93, the top 10 related diseases predicted by FCMDAP are listed in Table 7. Among the 10 diseases, eight were confirmed to be related to hsa-mir-93 by dbDEMC or PhenomiR databases. Adrenocortical carcinoma, which ranked 8, was not confirmed by these two databases. Heart failure, which ranked 1, was confirmed to be related to hsa-mir-93 in the literature. Ke et al. [49] found that miR-93 is related to cardiomyocyte apoptosis, and miR-93 can prevent cardiomyocyte apoptosis induced by myocardial ischemia/reperfusion by inhibiting PI3K/AKT/PTEN signaling.

**Table 7:** The top 10 diseasesrelated with hsa-mir-93 predicted by FCMDAP and their evidences

Rank	miRNA	Evidence
1	Heart Failure	PMID:27119510
2	Colonic Neoplasms	PhenomiR, dbDEMC
3	Carcinoma, Squamous Cell	PhenomiR, dbDEMC
4	Leukemia, Lymphocytic, Chronic, B-Cell	PhenomiR
5	Mesothelioma	dbDEMC
6	Pancreatic Neoplasms	PhenomiR, dbDEMC
7	Hodgkin Disease	dbDEMC
8	Adrenocortical Carcinoma	unconfirmed
9	Glioblastoma	PhenomiR, dbDEMC
10	Leukemia, Myeloid, Acute	PhenomiR, dbDEMC

## Discussion

In this work, we developed FCMDAP to predict human disease-related miRNAs. FCMDAP calculates the similarity between miRNAs by using mutual information based on the known miRNA-mRNA interaction information and adds the miRNA family information to construct a miRNA space. FCMDAP integrates disease functional similarity based on the disease-gene interaction and disease semantic similarity based on the DAG from MeSH to construct a disease space. FCMDAP integrates the association scores between miRNA and disease from miRNA and disease spaces. The association scores between miRNA and disease are calculated based on the *k* most similar neighbor recommendation algorithm, and miRNA cluster information is added into miRNA space. Like NSIM and other method, FCMDAP also predict unknown associations by constructing miRNA network and disease network. However, in the process, the similarity calculation process of miRNA and disease are independent of each other. Multiple types of data including miRNA-mRNA interaction, miRNA family information, disease-gene interaction, DAG from MeSH to calculate miRNA similarity, and disease similarity are considered and the prediction does not only depend on the known miRNA–diseases associations, thereby improving the accuracy of similarity calculations. Using the *k* most similar neighbor recommendation algorithm and miRNA cluster information makes the prediction results more reasonable, and improves the predictive performance.

LOOCV and case research show that FCMDAP exhibits excellent performance in predicting miRNA–disease associations. FCMDAP shows satisfactory performance in predicting diseases without any related miRNA information and miRNAs without any related disease information. The average AUC of FCMDAP for predicting isolated diseases and isolated miRNAs are 0.8417 and 0.8944, respectively. For isolated lung neoplasms, the prediction accuracy reached 100% in the top 50 predicted miRNAs. For the isolated hsa-mir-93, the prediction accuracy reached 90% in the top 10 diseases.

However, FCMDAP presents the following limitations. miRNA similarity can be further improved if other biomolecules that interact with miRNAs can be considered. As FCMDAP is developed on experimentally verified miRNA–disease associations, miRNA–disease associations can be experimentally verified, thereby improving the performance of FCMDAP.

## Conclusion

In order to provide effective support for experimental research on miRNAs, we proposed a computational method FCMDAP to find potential disease-related miRNAs. FCMDAP exhibits excellent performance in predicting potential disease-related miRNAs. The FCMDAP could extend to study on other biomeolecular networks and help to decipher the study of complex human disease pathogenesis and diagnosis.

## Additional files

Additional file 1: Known miRNA-disease associations. (XLSX 146 kb)

Additional file 2: Integrated disease similarity. (XLSX 1379 kb)

Additional file 3: Integrated miRNA similarity. (XLSX 1850 kb)

## Abbreviations

AUC: Area under the curve
DAG: Disease directed acyclic
LOOCV: Leave-one-out cross validation
MI: Mutual information
